# Effect of Holmium Oxide Loading on Nickel Catalyst Supported on Yttria-Stabilized Zirconia in Methane Dry Reforming

**DOI:** 10.1021/acsomega.2c04320

**Published:** 2022-11-21

**Authors:** Ahmed Sadeq Al-Fatesh, Ahmed A. Ibrahim, Anis H. Fakeeha, Fahad Albaqi, Khalid Anojaidi, Ibrahim Albinali, Ahmed E. Abasaeed, Francesco Frusteri, Sofiu L. Mahmud, Jehad K. Abu-Dahrieh, Abdulaziz A. Bagabas

**Affiliations:** † Chemical Engineering Department, College of Engineering, King Saud University, P.O. Box 800, Riyadh11421, Saudi Arabia; ‡ National Petrochemical Technology Center (NPTC), Materials Science Research Institute (MSRI), King Abdulaziz City for Science and Technology (KACST), P.O. Box 6086, Riyadh11442, Saudi Arabia; § CNR-ITAE, Istituto di Tecnologie Avanzate per Energia “Nicola Giordano”, Via S. Lucia sopra Contesse 5, 98126Messina, Italy; ∥ School of Chemistry and Chemical Engineering, Queen’s University Belfast, BelfastBT9 5AG, U.K.

## Abstract

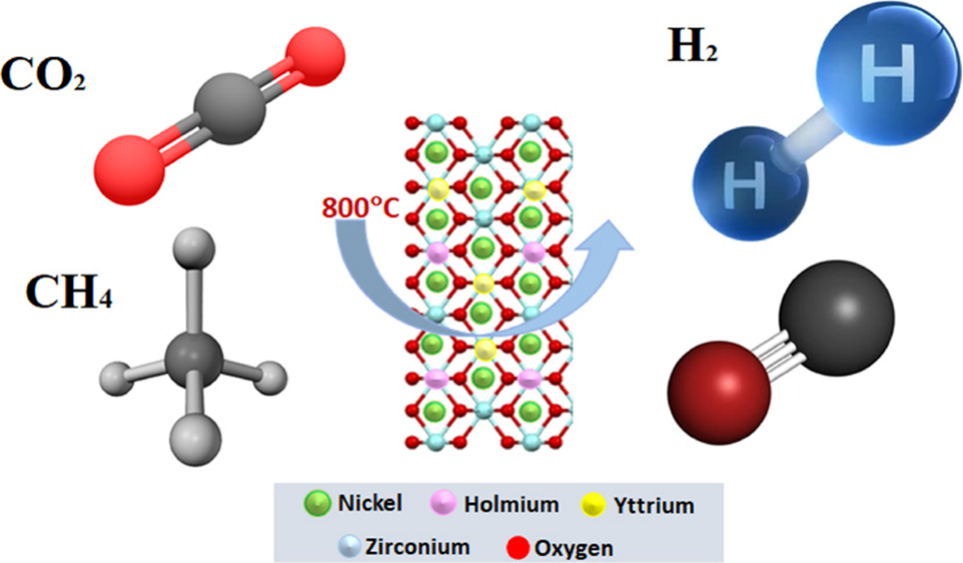

The carbon dioxide reforming of methane has attracted attention from researchers owing to its possibility of both mitigating the hazards of reactants and producing useful chemical intermediates. In this framework, the activity of the nickel-based catalysts, supported by yttria-stabilized zirconia and promoted with holmium oxide (Ho_2_O_3_), was assessed in carbon dioxide reforming of methane at 800 °C. The catalysts were characterized by N_2_-physisorption, H_2_ temperature-programmed reduction, temperature-programmed desorption of CO_2_, X-ray diffraction, scanning electron microscopy (SEM) together with energy-dispersive X-ray spectroscopy, transmission electron microscopy (TEM), and thermogravimetric analysis (TGA) techniques. The effect of holmium oxide weight percent loading (0.0, 1.0, 2.0, 3,0, 4.0, and 5.0 wt %) was examined owing to its impact on the developed catalysts. The optimum loading of Ho_2_O_3_ was found to be 4.0 wt %, where the methane and carbon dioxide conversions were 85 and 91%, respectively. The nitrogen adsorption–desorption isotherms specified the mesoporous aspect of the catalysts, while the SEM images displayed a morphology of agglomerated, porous particles. The TEM images of the spent catalyst displayed the formation of multiwalled carbon nanotubes. TGA of the 4.0 wt % of Ho_2_O_3_ catalyst, experimented over 7-hour time-on-stream, displayed little weight loss (<14.0 wt %) owing to carbon formation, indicating the good resistance of the catalyst to carbon accumulation due to the enhancing ability of Ho_2_O_3_ and its adjustment of the support.

## Introduction

The global energy demand escalates due to the depletion of fossil fuels.^1^ The world is suffering from environmental pollution as a result of gas emissions of carbon dioxide (CO_2_) and methane (CH_4_), which are included in the set of greenhouse gases responsible for global warming.^2−5^ Consequently, developing a strategy to mitigate the carbon impact and promote the availability of clean ambient and alternative energy sources is of paramount importance for the current research.^6^ Thus, CO_2_ reforming of CH_4_ (CRM) has attracted immense attention from researchers owing to its capability of decreasing the deterioration effects of CO_2_ and CH_4_ and generating clean fuels. Generally speaking, different reforming techniques are employed for CH_4_ valorization. For instance, steam reforming of methane (SRM)^7−10^ and partial oxidation of methane^11,12^ are extensively investigated. The CRM, which is carried out at high temperatures owing to the endothermicity of the reaction (eq 1), generates synthesis gas.^13−15^
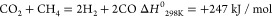
1

The produced synthesis gas (H_2_ and CO) develops a ratio close to unity, which favors the Fischer–Tropsch reaction and oxygenated chemicals.^16,17^ The syngas by CRM is also affected by the parallel reverse water–gas shift (RWGS) reaction (eq 2), where part of the produced H_2_ reacts with CO_2_ to give CO, which results in the decrease of the H_2_/CO ratio. The CRM exhibits side reactions of methane decomposition (eq 3) and Boudouard reaction (eq 4) at the proper temperature range. Eqs 3 and 4 enhance the carbon deposition on the surface of the catalyst.
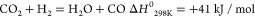
2
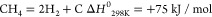
3
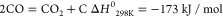
4

Many catalysts have been employed to improve the thermally catalytic activity and stability of the CRM process. Nobel metals such as Pd, Pt, Rh, and Ru were examined. Their scarcity and high prices make them inappropriate for large-scale production.^18−20^ Transition metals like Ni, Co, and Fe are often used due to their availability and low costs.^21,22^ Active nickel-based catalysts are widely used due to their best activity among these three metals. However, since CRM reactions are conducted at high temperatures, nickel-based catalysts are prone to fast deactivation as a result of active site sintering and carbon deposition.^23−25^ Several techniques have been suggested for upgrading the properties of Ni catalysts. These techniques include the use of suitable support and promoter because they interact with the active metal and influence the mechanism of carbon formation.^26^ Besides the adequate thermal stability and mechanical resistance, the support must possess low acidic properties to reduce the deposition of carbon, which leads to the deactivation of the catalyst.^27^ Zirconia (ZrO_2_) with Lewis basicity ranks among the best supports to inhibit carbon formation, as confirmed by the study of Yamazaki et al.^27^ The ZrO_2_ support has several phases with various oxygen mobility and different strengths of the metal–support interactions. These properties of zirconia could influence the thermal sintering of active metal particles and the elimination of deposited coke particles on the catalyst surface. The phase transformation can be inhibited by doping a certain amount of MgO, CaO, Sc_2_O_3_, Y_2_O_3_, or CeO_2_ into zirconia. For instance, cubic ZrO_2_ is strongly stabilized relative to monoclinic ZrO_2_ and tetragonal ZrO_2_ by the addition of yttria (Y_2_O_3_).^28^ The stabilization of ZrO_2_ with yttria improves the oxygen storage, transport properties, and thermal resistance of the catalyst. The catalytic activity of ZrO_2_ could be also enhanced by the addition of other metal oxide dopants such as MoO_3_, stabilizing the monoclinic phase of zirconia particularly, for the hydrodesulfurization process.^29^ Charisiou and co-authors investigated the role of a Ni catalyst, supported over ZrO_2_ stabilized with yttria, in the glycerol SRM.^30^ Their findings displayed the intensification of the O_2_ storage size of the support and the dominance of moderate strength acid sites of the catalyst. The modified support presented more stable monodentate carbonates. Moreover, the catalyst was depicted by notably smaller Ni particles and larger Ni surface concentrations. These aspects altered the product scattering by enhancing the selectivity and yield of H_2_ and by obstructing the conversion of CO_2_ to CO. Bahari et al. elaborated on the impact of Y_2_O_3_ promoter doping on CRM over the Co/mesoporous alumina (Co/m-Al_2_O_3_) catalyst.^31^ Their results displayed that the Co particle dispersion on m-Al_2_O_3_ was markedly enhanced after promoter addition, causing the reduction in crystallite size and Co agglomeration. Furthermore, the Y_2_O_3_ promoter decreased catalyst reducibility by strengthening the interaction between Co metal and the m-Al_2_O_3_ support. The YCo/m-Al_2_O_3_ catalyst yielded the highest activity and the minimum carbon deposition owing to the excessive Co dispersion, small Co particle size with strong Co/mesoporous alumina interaction, and greater oxygen storage capacity. On the other hand, the incorporation of promoters leads to a number of advantageous properties such as better metal dispersion, higher Brunauer–Emmett–Teller (BET) specific surface area, superior reducibility, and hence improved efficacy toward CRM. The addition of promoters not only adds new active sites, but also can intensify nickel dispersion^32^ or resistance to coking by augmenting the basicity^33^ and increasing the oxygen storage capacity. Amin et al. examined the role of lanthanide promoters such as holmium oxide (Ho_2_O_3_) on Ni/Al_2_O_3_ catalysts in CRM.^34^ They obtained outstanding stability for the promoted Ni/Al_2_O_3_ catalysts due to the lower quantities of amorphous carbon deposition in comparison to the un-promoted catalyst. Duan and co-authors investigated the denitration through ammonia selective catalytic reduction, where the presence of potassium oxide in the exhaust gas had a deactivation effect on catalysts.^35^ They found that the addition of holmium oxide on the Ce-Ti oxide catalyst provided more Lewis acid sites and could notably nurture its potassium tolerance. Fakeeha et al. studied the effect of a narrow range of holmium oxide promoter loadings on Ni-supported zirconia, employed for CRM.^36^ They found that Ho_2_O_3_ improved the basicity of the catalyst and, therefore, boosted the chemisorption and activation of CO_2_, which consecutively reduced the coke formation.

In this article, we developed Ho_2_O_3_-promoted Ni-based catalysts, supported on 8% yttria-stabilized zirconia, for dry reforming of methane. Different loadings of Ho_2_O_3_ (0.0, 1.0, 2.0, 3.0, 4.0, and 5.0 wt %) were investigated to evaluate their effects on the catalyst characters and hence on their overall performance.

## Experimental Section

### Materials

All chemicals were acquired commercially and were experimented without further purification. Holmium nitrate pentahydrate [Ho (NO_3_)_3_^.^5H_2_O, 99.999% trace metals basis] was obtained from Sigma-Aldrich, while nickel nitrate hexahydrate [Ni (NO_3_)_2_^.^6H_2_O, 98%] and mesoporous 8.0 wt % yttria-stabilized zirconia (*meso*-8Y_2_O_3_-ZrO_2_; *meso*-YZr) were purchased from Alfa Aesar. To produce ultrapure water (18.2 MΩ.cm), a Milli-Q water purification system (Millipore, Burlington, MA, USA) was employed.

### Catalyst Synthesis

The required amounts of Ni(NO_3_)_2_^.^6H_2_O to give 5.0 wt % loading of NiO (98%, Alfa Aesar), Ho(NO_3_)_3_^.^5H_2_O to give 0.0, 1.0, 2.0, 3.0, 4.0, or 5.0 wt % loading of Ho_2_O_3_, and *meso*-YZr support were ground mechanically together by using mortar and pestle. Ultrapure water was then added by a droplet to form a paste with the solid mixture, followed by mechanical mixing until a dried solid mixture was attained. Wetting and drying processes of the solid mixture were repeated three times.

The solid products were subsequently weighed and calcined at 600 °C for a period of 3 hours. The catalysts were referred to in this paper as Ni-xHo-YZr (*x* = 0.0, 1.0, 2.0, 3.0, 4.0, and 5.0).

### Sieve Analysis

The particle size of the catalysts was measured by passing the materials through a series of sieves stacked with progressively smaller openings from top to bottom, where the material retained on each sieve was collected. The meshes used for sieving had sizes of 0.71, 0.5, 0.3, and 0.15 mm.

### Catalyst Characterization

The techniques for characterization, used during these experiments, comprised nitrogen physisorption, temperature-programmed desorption of CO_2_ or H_2_ (CO_2_-TPD and H_2_-TPD), H_2_ temperature-programmed reduction (H_2_-TPR), powder X-ray diffraction (XRD), thermogravimetric analysis, transmission electron microscopy (TEM), and field emission scanning electron microscopy (SEM) together with energy-dispersive X-ray spectroscopy (EDX). A comprehensive description of the equipment and the method of characterization are given in the Supporting Information. Likewise, the description of the catalyst efficiency is provided there.

## Results and Discussion

**Figure 1:**
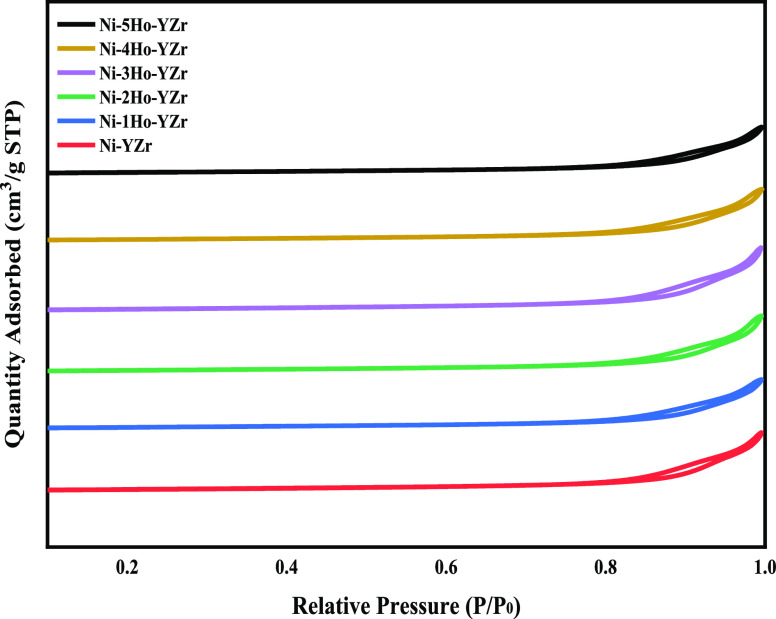
Nitrogen adsorption–desorption isotherms for the Ni-supported catalysts.

To comprehend the performance variations of the prepared catalysts, nitrogen adsorption–desorption isotherms of the catalysts were recorded for examining the textural features: specific surface area (BET), pore volume (*P*_v_), and pore size (*P*_s_). Figure 1 displays the nitrogen adsorption–desorption isotherms, which denoted the mesoporosity aspect of the catalysts as the isotherms were of type IV with a hysteresis loop of the H3-type according to the IUPAC classification. A H3-type hysteresis loop suggests that they are slit pores indicating the presence of nonuniform, slit-shaped pores with parallel walls. This type is often found with micromesoporous carbon.^37^ Doping with the active metal and promoter did not alter the framework of the support. However, a considerable rise in the relative pressure region of 0.65–0.95 was observed because of the unification of N_2_ capillary condensation within the mesopores. The physisorption results are exhibited in Table 1. The nickel oxide supported on holmium oxide-promoted yttria-stabilized zirconia showed lower BET specific surface areas than the nickel oxide supported on un-promoted yttria-stabilized zirconia. The addition of holmium oxide obstructed the pore of the zirconia and hence reduced the BET specific surface areas and pore volume.

**Table 1:** N_2_-Physisorption Results of the Catalysts

sample	BET (m^2^/g)	pore volume (g/cm^3^)	pore size (nm)
YZr	62.6	0.16	23.9
Ni-YZr	30.8	0.19	24.8
Ni-1Ho-YZr	28.1	0.16	23.5
Ni-2Ho-YZr	29.2	0.18	24.9
Ni-3Ho-YZr	33.3	0.21	25.1
Ni-4Ho-YZr	28.1	0.16	23.7
Ni-5Ho-YZr	25.7	0.15	23.7

**Figure 2:**
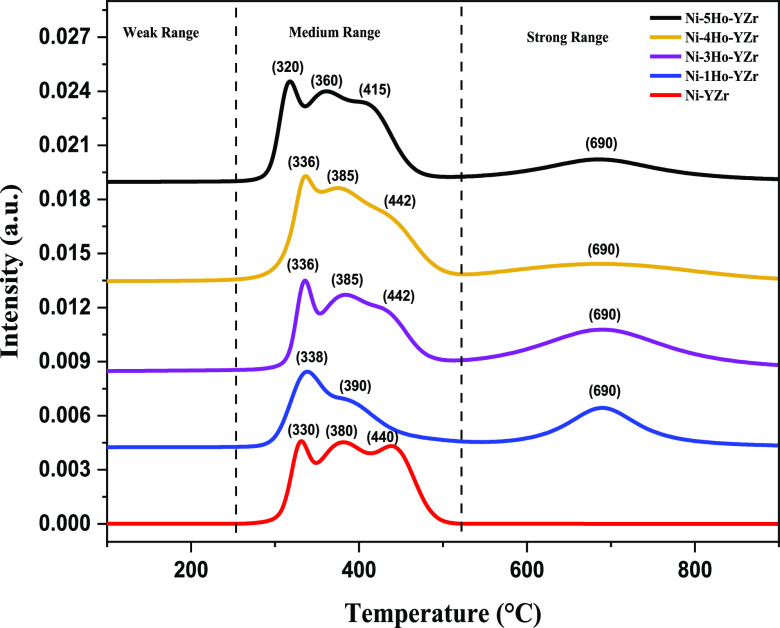
H_2_-TPR profiles for the Ni-xHo-YZr (*x* = 0, 1, 3, 4, and 5) catalysts.

**Figure 3:**
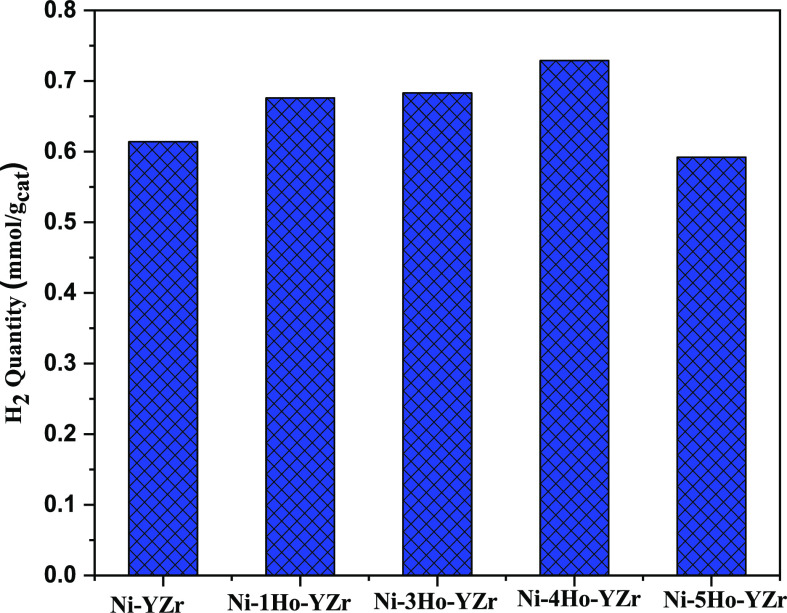
Quantity of hydrogen consumption during TPR analysis.

The H_2_-TPR profiles of the fresh catalysts, Ni-*x*Ho-YZr (*x* = 0, 1, 3, 4, and 5), are shown in Figure 2. All the reduction peaks were detected above 300 °C and below 1000 °C; furthermore, all of the peaks were in the middle-temperature range of 300–500 °C, except one broad peak in the high-temperature range of 600–800 °C for the Ho_2_O_3_-promoted catalysts, while the un-promoted catalyst displayed only three peaks in the middle-temperature range at around 330, 380, and 440 °C. These are attributed to the reduction of nickel oxide (NiO → Ni) with varying interactions with YZr support. Besides the overlapping peaks of Y and Zr particles, such an observation indicated that the Ho_2_O_3_ promoter strengthened the interaction between NiO and YZr support. In addition, the absence of reduction peaks below 300 °C implied that there were no free NiO or weakly interacted species with YZr support. For the Ho_2_O_3_-promoted catalysts (Ho_2_O_3_ = 1.0–4.0 wt %), the three reduction peaks in the middle-temperature range were shifted toward higher temperatures, implying stronger interaction between NiO and YZr support with increasing the amount of the Ho_2_O_3_ promoter. Furthermore, a peak in the high-temperature range was evolved with impregnating the Ho_2_O_3_ promoter in the texture of the catalyst owing to the increase in the interaction between NiO and YZr support. However, the intensity of this peak was decreased with increasing the Ho_2_O_3_ amount from 1.0 to 4.0 wt %, indicating the reduction in the interaction strength of NiO with YZr. The weakest interaction that occurred between NiO and YZr, when the Ho_2_O_3_ amount was 4.0 wt %, facilitated the reduction of NiO to Ni metal, which is the active site for abstracting hydrogen from methane and hence made it the best catalyst. On the other hand, increasing the Ho_2_O_3_ amount to 5.0 wt % shifted the three peaks in the middle-temperature range to lower temperatures than those of the other catalysts and, at the same time, increased the intensity of the peak in the high-temperature range than that in the case of 4.0 wt % Ho_2_O_3_. Such observations would help the formation of agglomerated Ni metal species, resulting in the reduction in the activity of Ni-5Ho-YZr in comparison to the other promoted catalysts. Figure 3 displays the H_2_ consumption quantities of the Ni-xHo-YZr (*x* = 0, 1, 3, 4, and 5) catalysts. It showed that by increasing the loading of Ho_2_O_3_ from 0.0 to 4.0 wt %, the H_2_ consumption quantities increased, which led to a higher dispersion of nickel oxide on the surface. These results were in agreement with the observed methane conversion upon increasing the Ho_2_O_3_ promoter from 0.0 to 4.0 wt %.

**Figure 4:**
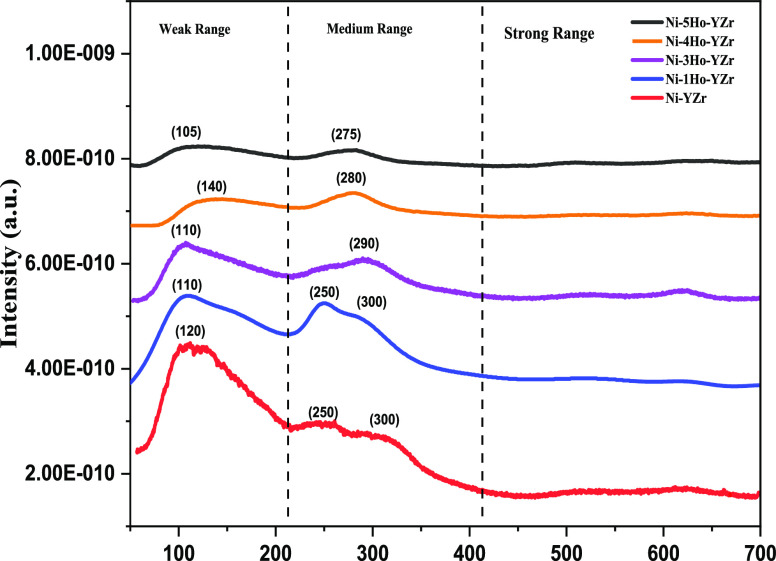
CO_2_-TPD profiles for the Ni-xHo-YZr (*x* = 0, 1, 3, 4, and 5) catalysts.

Figure 4 displays the CO_2_-TPD profiles for estimating the catalyst surface basicity. Increasing the loading of Ho_2_O_3_ from 0.0 to 5.0 wt % led to the decrease in peak intensities of CO_2_ desorption, that is, reduction in surface basicity. One broad peak in the low-temperature range of 50–200 °C, with a maximum of ∼120 °C, was observed for the un-promoted catalyst, indicating the weak basic sites. Increasing the content of Ho_2_O_3_ to 1.0, 3.0, and 5.0 wt % shifted the center of this peak to lower temperature, viz., weakened the basic sites more than those of the un-promoted catalyst. The 4.0 wt % Ho_2_O_3_, on the contrary, caused a shift to a higher temperature for the maximum of this peak (∼140 °C). On the other hand, two broad peaks, with a maximum of 250° and 300 °C, were detected in the middle-temperature range, 200–400 °C, for medium basic sites on the surface of the un-promoted catalyst. These two peaks had almost the same positions in the temperature scale when Ho_2_O_3_ content was 1.0 wt %, but with higher intensities. Increasing the Ho_2_O_3_ content to 3.0, 4.0, and 5.0 wt % overlapped these two peaks and shifted the maximum to higher temperatures (290, 280, and 275 °C), corresponding to 3.0, 4.0, and 5.0 wt % Ho_2_O_3_ content, implying stronger adsorption of CO_2_ in the middle-temperature range. All these observations were in agreement with the acidity nature of Ho_2_O_3._^38^

**Figure 5:**
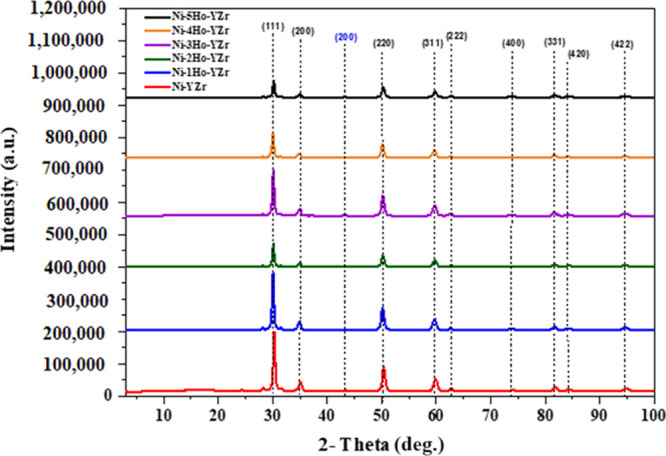
XRD patterns belong to cubic zirconia of the fresh Ni-xHo-YZr (*x* = 0.0, 1.0, 2.0, 3.0, 4.0, or 5.0 wt %) catalysts.

Figure 5 displays the XRD patterns of the fresh Ni-xHo-YZr (*x* = 0.0, 1.0, 2.0, 3.0, 4.0, or 5.0 wt %) catalysts, where the black label is for the yttria–stabilized cubic zirconia (JCPDS No. 49-1642), while the blue label is for the cubic nickel oxide (PDF 00-044-1159). Table 2 exhibits the shift in the 2θ angle and the change in the d-spacing of (111) and (220) crystallographic planes of the yttria-stabilized cubic zirconia phase for the fresh catalysts. The addition of a holmium promoter moved the peaks of YZr support slightly to a smaller 2θ angle, that is, produced a slight increase in the d-spacing parameter, indicating the incorporation of Ho_2_O_3_ in the lattice of the YZr support, as displayed in Table 2. Scherrer’s formula is employed for the computation of the nanocrystallite size in the form of powder, which can be denoted as follows:
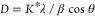


**Table 2:** Shift in the 2θ Angle and the Change in the d-Spacing of (111) and (220) Crystallographic Planes of the Yttria-Stabilized Cubic Zirconia Phase for the Fresh Catalysts

catalyst	Ho_2_O_3_ wt %	2θ (°)	d-spacing for (111), Å	2θ (°)	d-spacing for (220), Å	average crystallite size, nm
Ni-YZr	0.0	30.15761	2.96100	50.27839	1.81324	20.4
Ni-1Ho-YZr	1.0	30.14662	2.96205	50.25926	1.81388	21.00
Ni-2Ho-YZr	2.0	30.10567	2.96599	50.18793	1.81629	21.19
Ni-3Ho-YZr	3.0	30.09574	2.96695	50.17064	1.81688	20.93
Ni-4Ho-YZr	4.0	30.07358	2.96908	50.13207	1.81818	19.20
Ni-5Ho-YZr	5.0	30.05972	2.97042	50.10793	1.81900	20.85

where *D* is the crystallite size in nanometers, *K* is the shape factor which is 0.94, λ is the wavelength of X-ray, β is the full width at half maximum of the diffraction peak of the sample, and θ is the Bragg diffraction angle in degrees. The crystallite sizes of different Ho_2_O_3_ loadings in Ni-xHo-YZr (*x* = 0.0, 1.0, 2.0, 3.0, 4.0, or 5.0 wt %) catalysts were determined from XRD by using the most intense peaks of (111) and (220).

**Figure 6:**
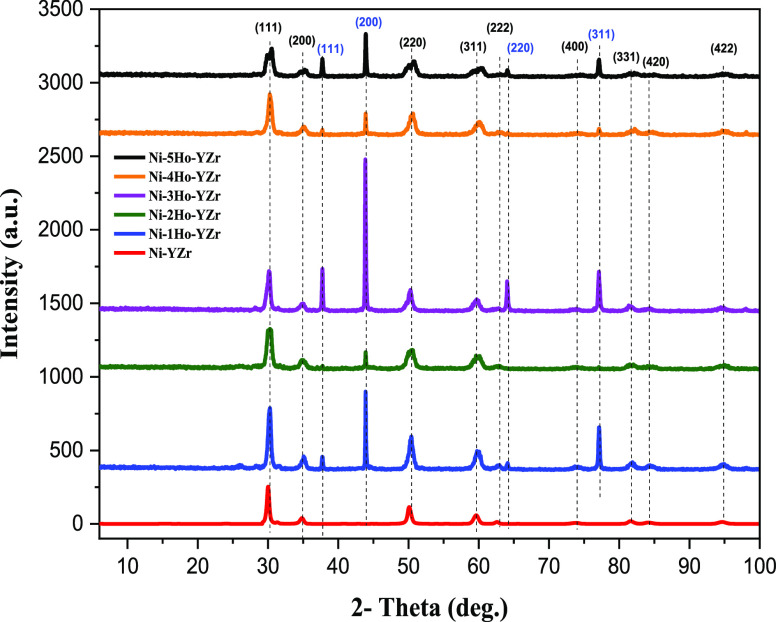
XRD patterns belonging to cubic zirconia of the spent Ni-xHo-YZr (*x* = 0.0, 1.0, 2.0, 3.0, 4.0, or 5.0 wt %) catalysts.

Figure 6 displays the XRD patterns of the spent Ni-xHo-YZr (*x* = 0.0, 1.0, 2.0, 3.0, 4.0, or 5.0 wt %) catalysts. Table 3 exhibits the shift in the 2θ angle and the change in the d-spacing of (111) and (220) crystallographic planes of the yttria-stabilized cubic zirconia phase for the spent catalysts. The addition of a holmium promoter moved the peaks of YZr support slightly to a greater 2θ angle, that is, produced a slight decrease in the d-spacing parameter, indicating the expelling of Ho_2_O_3_ out of the lattice of the YZr support, as displayed in Table 3. The crystallite sizes of the spent Ni-xHo-YZr (*x* = 0.0, 1.0, 2.0, 3.0, 4.0, or 5.0 wt %) catalysts were assessed. We observed that the crystallite size of the spent catalysts was decreased in comparison to those of the fresh catalysts because the Ho_2_O_3_ promoter might segregate from the support.

**Table 3:** Shift in the 2θ Angle and the Change in the d-Spacing of (111) and (220) Crystallographic Planes of the Yttria-Stabilized Cubic Zirconia Phase for the Spent Catalysts

catalyst	Ho_2_O_3_ wt %	2θ (°)	d-spacing for (111), Å	2θ (°)	d-spacing for (220), Å	average crystallite size, nm
Ni-YZr	0.0	30.00	2.9764	50.04	1.8214	20.63
Ni-1Ho-YZr	1.0	30.15	2.9610	50.05	1.8211	17.17
Ni-2Ho-YZr	2.0	30.25	2.9518	50.15	1.8177	10.54
Ni-3Ho-YZr	3.0	30.26	2.9510	50.16	1.8171	15.34
Ni-4Ho-YZr	4.0	30.36	2.9415	50.27	1.813	13.45
Ni-5Ho-YZr	5.0	30.39	2.9388	50.67	1.8000	7.73

**Figure 7:**
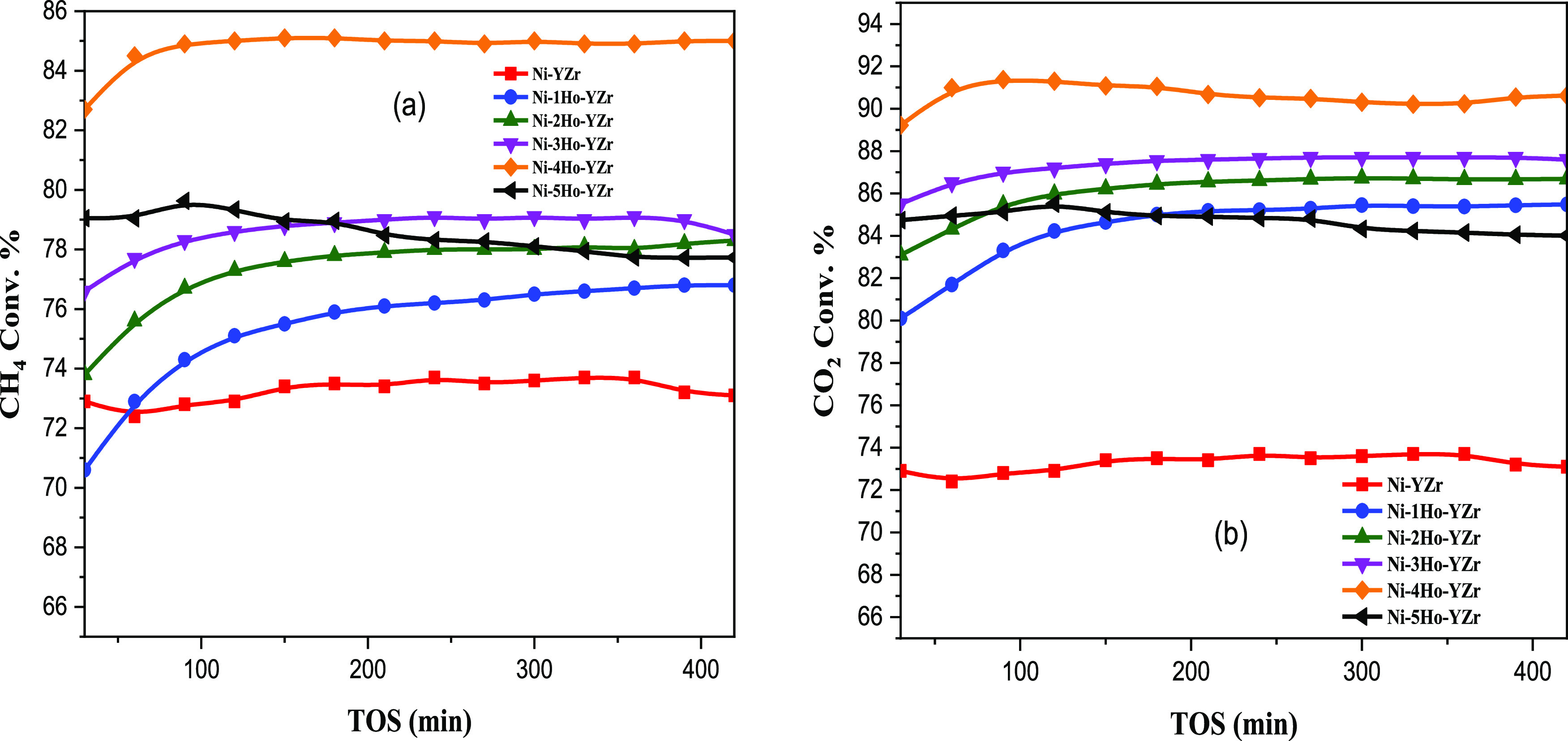
Activity comparison between the un-promoted Ni-YZr catalyst and the promoted samples: (calcination = 600 °C, activation = 800 °C, reaction = 800 °C). (a) CH_4_ conversion and (b) CO_2_ conversion.

The suitability of Ni-xHo-YZr (*x* = 0.0, 1.0, 2.0, 3.0, 4.0, or 5.0 wt %) catalysts for DRM was assessed at 800 °C. Figure 7a displays the stability test for the conversion of CH_4_, run for 420 min. The doping with the Ho_2_O_3_ promoter boosted the average CH_4_ conversion upon increasing the Ho_2_O_3_ loading from 0.0 to 1.0, 2.0, 3.0, and 4.0 wt % to reach 73, 76, 78, 79, and 85%, respectively. However, a further increase of Ho_2_O_3_ loading to 5.0 wt % reduced the average CH_4_ conversion to 77%. Such conversion could be due to the synergetic effect of the active catalyst of nickel and the Ho_2_O_3_ promoter at loadings in the range of 1.0–4.0 wt %, which, in turn, enhanced the dispersion by increasing the chances of CH_4_ gas adsorption on the surface. In contrast, the loading of 5.0 wt % Ho_2_O_3_ covered partially the active nickel surface and hence lowered the activity. The Ni-4Ho-YZr catalyst gave the best achievment in activity and stability. Figure 7b displays the stability test for the conversion of CO_2_, carried out for 420 min at 800 °C. The CO_2_ conversion profile increased with increasing the Ho_2_O_3_ promoter loading from 0.0 to 4.0 wt % and reduced after increasing it to 5.0 wt %. Such behavior had the same observed trend in the reducibility of nickel oxide (H_2_-TPR, Figure 2) with increasing the promoter loading. Nevertheless, the CO_2_ conversion was always greater than that of CH_4_. This occurrence is ascribed to the effect of the RWGS reaction (eq 2), where CO_2_ is additionally taken by the hydrogen in the product. This conclusion about the functioning of RWGS was supported by plotting the H_2_/CO mole ratio against time-on-stream (TOS), as shown in Figure S1. Increasing the loading of the Ho_2_O_3_ promoter (0.0–4.0 wt %) led to the increasing hydrogen production, as reflected by the increasing H_2_/CO mole ratio from 0.87 to 0.97 owing to the increasing catalyst surface acidity (vide supra Figure 4). However, increasing the Ho_2_O_3_ promoter loading to 5.0 wt % resulted in the reduction of the H_2_/CO mole ratio to 0.91 due to the reduction in catalytic activity of methane decomposion.

In general, the catalysts showed stable catalytic activity during the reaction course of 420 min. Thus, the conversion profiles of both CH_4_ and CO_2_ are assumed to be rather diminished with TOS owing to the consequences of side reactions of eqs 3 and 4, where carbon formation on the catalyst was attained, as indicated by the TGA results of Figure S4. The DRM performance of the present work contrasted with those in the past with regard to methane conversion. The result shown in Table 4 depicts the worthiness of our catalyst.

**Table 4:** Contrast between our Obtained CH_4_ Conversion and Those Accomplished in the Past for Different Catalysts Applied to DRM^a^

catalyst	GHSV mlg^–1^ h^–1^	cat. wt., mg	reaction temp., °C	CH_4_ conv., %	ref.
5%Ni + 5%Ce/La_2_O_3_ + ZrO_2_	42,000	100	700	83	(39)
15%Ni/Al_2_O_3_ + 15%ZrO_2_	30,000	100	700	84	(40)
Ni@ZrO_2_-SiZr-6.1	72,000	100	800	82	(41)
10%Ni + 3%Co/Al_2_O_3_-ZrO_2_-U(T20-P30)	24,000	100	750	80	(42)
Ni + Zr_2_O_3_/SiO_2_-C	48,000	200	800	82	(43)
Ni-4Ho-YZr	42,000	100	800	85	present

a CH_4_: CO_2_ mole ratio = 1:1.

It has been reported in the literature that zirconia (ZrO_2_) facilitates the dissociation of carbon dioxide into carbon monoxide and oxygen radical owing to the oxygen vacancy in zirconia support, as illustrated in eq 5:^44^
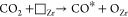
5

Furthermore, carbon monoxide might be formed by the dissociation of bicarbonate intermediate, as shown in eqs 6 and 7
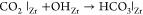
6
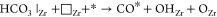
7□_Zr_ represents an oxygen vacancy on the zirconia surface, while O_Zr_, and OH_Zr_ are surface oxygen and hydroxyl species, respectively.

Stabilizing zirconia by yttria increases the number of oxygen vacancies, provides basic sites, and hence improves the dissociation of carbon dioxide,^45^ as per eq 5. The addition of holmium oxide (Ho_2_O_3_) would also contribute to the enhancement of the adsorption of carbon dioxide via its binding to Ho^3+^ ions and the formation of radical carbonate anions, which, in turn, facilitates the activation of terminal C=O bonds.^46^

The adsorbed oxygen radical would react with the products of methane decomposition, that is, carbon and hydrogen. Thus, it is useful for the removal of coke carbon from the surface of nickel metal,^44^ as shown in eq 8
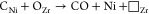
8Where C_Ni_ is carbon deposited on the surface of nickel metal.

In addition, the oxygen radical could facilitate the dissociation of methane by reacting with the CH_*x*_ groups, formed by the abstraction of hydrogen from methane over the surface of nickel metal, as shown in eqs 9 and 10
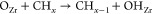
9
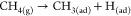
10Where CH_3(ad)_ and H_(ad)_ are adsorbed species on the surface of nickel metal.

The components of our catalysts are zirconia, yttria, holmium oxide, and nickel metal, where the oxides are the majors. Therefore, the most probable route for the dry reforming of methane over our catalysts is the promotion of methane decomposition by the dissociation of carbon dioxide, as illustrated in the chemical eqs 5–10.

**Figure 8:**
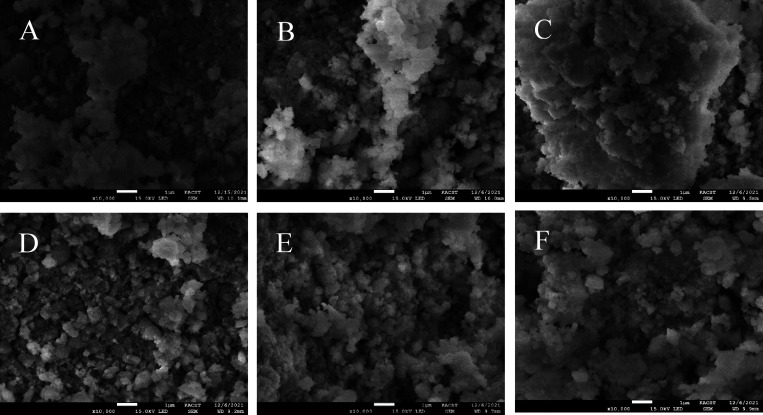
SEM images of the fresh catalysts: (A) Ni-0Ho-YZ, (B) Ni-1Ho-YZ, (C) Ni-2Ho-YZ, (D) Ni-3Ho-YZ, (E) Ni-4Ho-YZ, and (F) Ni-5Ho-YZ.

Figure 8 shows the SEM images for all the fresh catalysts, where the same morphology of agglomerated, undefined shape particles was observed irrespective of the Ho_2_O_3_ promoter content.

**Figure 9:**
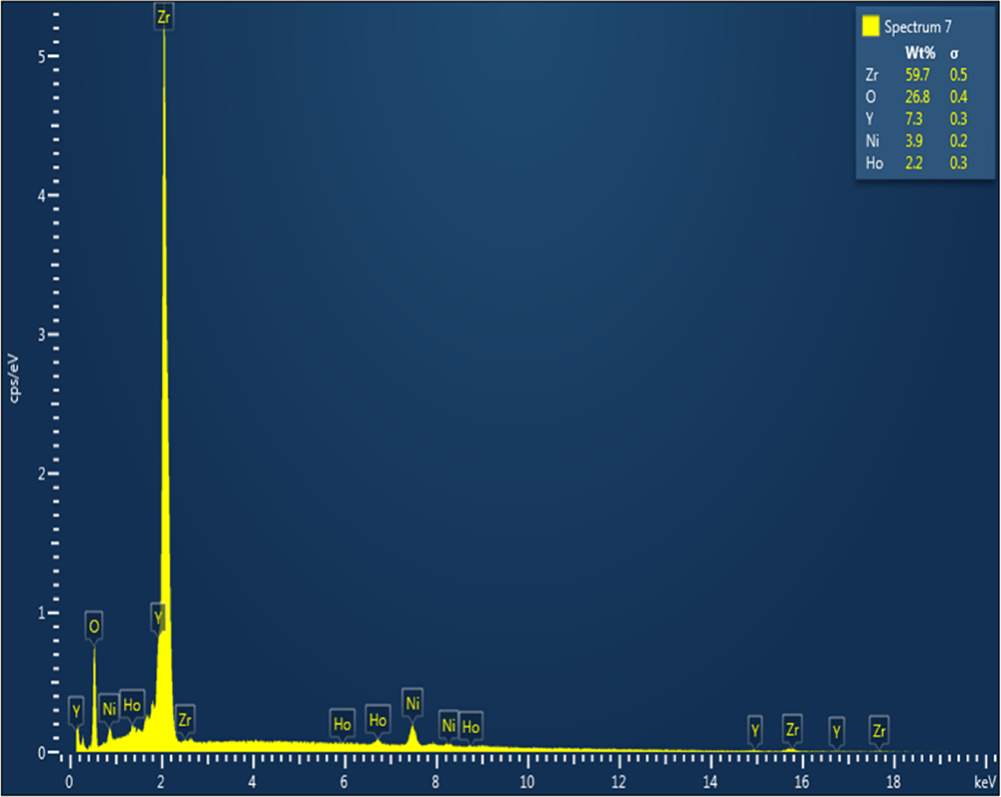
EDX of the fresh Ni-4Ho-YZ catalyst.

Figure 9 displays the EDX analysis for a fresh sample of the best catalyst of Ni-4Ho-YZ, where all the expected elements to be on the surface were detected qualitatively. However, in terms of quantitative analysis, this surface analysis showed that the experimental surface value for nickel was 3.9%, which was identical to the theoretical one (3.9%), implying that all the active catalysts of nickel resided on the surface for complete utilization of nickel in the CRM process. The experimental surface content of zirconium (59.7%) and oxygen (26.8%) was very close to their corresponding theoretical values of ∼62 and ∼25%, respectively. Such findings were in agreement with enhancing the surface basicity of the catalyst. The experimental yttrium surface content of 7.3% was higher than the theoretical value of 5.7%. This high surface yttrium content would also boost the surface basicity and oxygen storage, improve resistance to coke deposition, and imply that yttrium substituted zirconium in the lattice of zirconia.^47^ On the other hand, the surface content of the holmium promoter of 2.2% was lower than the theoretical value of 3.5%, implying that most holmium was not present on the surface, but rather was embedded in the catalyst bulk. All these surficial features were in agreement with the observed efficiency of the Ni-4Ho-YZ catalyst.

**Figure 10:**
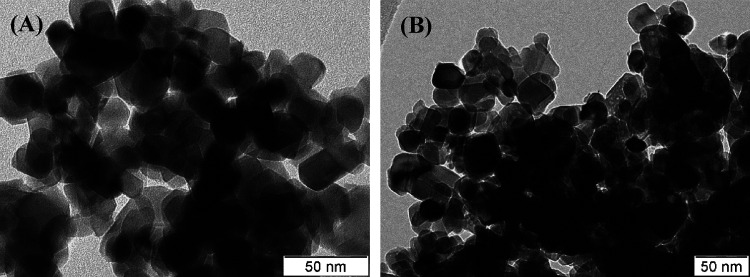
TEM images of the fresh catalysts: (A) Ni-YZ and (B) Ni-4Ho-YZ.

**Figure 11:**
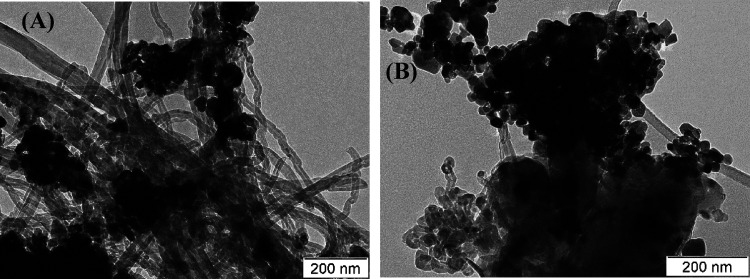
TEM images of spent catalysts (A) Ni-YZ and (B) Ni-4Ho-YZ.

Table 5 displays the surface elemental contents of all catalysts. The highest nickel content was observed for the Ni-0Ho-YZr catalyst, implying that nickel oxide was agglomerated on the surface and forming large particles with a weak interaction with the support. On the other hand, the lowest nickel content was for the Ni-4Ho-YZr, indicating that nickel oxide was dispersed and interacted with the support surface. Such results could be correlated to the observed activity in terms of methane and carbon dioxide conversions as well as the H_2_-TPR results. The highest holmium content was obtained for the Ni-4Ho-YZr catalyst, while the lowest holmium was obtained for Ni-2Ho-YZr. Once again, increasing the holmium content on the surface would increase the reducibility of catalysts and reduce the carbon deposition, as per the observed results of conversions. The highest yttrium content was observed for the Ni-4Ho-YZr catalyst among the promoted catalysts with Ho_2_O_3_ in the range of 1.0–5.0 wt % because the higher the yttrium content is, the higher is nickel dispersion and the higher is the activity too. However, the higher yttrium content than Ni-4Ho-YZr could not lead to a parallel performance due to the lack of holmium. All these observations support the observed results. Figure S2 shows the SEM images for all the spent catalysts, where filamentous carbon nanotubes on the surfaces of the catalysts were observed. The deposition of carbon nanotubes could be responsible for catalyst deactivation. The morphology of the spent catalysts resembled the fresh catalysts. Figure S3 displays the EDX analysis for a spent sample of the best catalyst of Ni-4Ho-YZ. It showed qualitatively the presence of all metal components over the surface of the catalysts besides carbon. Figures 10 and 11 show the TEM images of some fresh catalysts and their corresponding spent catalysts, respectively. Both fresh and spent catalysts had the same morphologies as those observed in the SEM images. However, multi-walled carbon nanotubes were observed in the spent catalysts due to carbon deposition during the course of the CRM reaction. The SEM and TEM images supported each other.

**Table 5:** Comparison of Theoretical and EDX Experimental Surface Elemental Contents of the Catalysts

	Ni-0Ho-YZr		Ni-1Ho-YZr		Ni-2Ho-YZr		Ni-3Ho-YZr		Ni-4Ho-YZr		Ni-5Ho-YZr	
	theo.	ex.	theo.	ex.	theo.	ex.	theo.	ex.	theo.	ex.	theo.	ex.
	wt %											
Ni	3.9	6.7	3.9	5	3.9	4.1	3.9	5	3.9	3.9	3.9	5
Ho	0	0	0.87	0.7	1.74	0.6	2.62	1.6	3.49	2.2	4.36	1.2
Y	6	7.4	5.92	7.1	5.86	7	5.8	7.2	5.73	7.3	5.67	7
Zr	64.7	62.9	64	58.6	63.34	57.4	62.66	58.9	61.98	59.7	61.3	59
O	25.37	23.1	25.28	28.5	25.13	30.9	24.99	27.3	24.87	26.8	24.74	27.8

For a quantitative estimation of the extent of carbon deposition on the spent Ni-Zr and Ni-xHo-YZr (*x* = 0.0, 1.0, 2.0, 3.0, 4.0, or 5.0) catalysts, TGA analysis was conducted, as shown in Figure S4. When the active catalyst of nickel was supported on ZrO_2_ stabilized with Y_2_O_3_, the carbon deposition was 19%. As the Ho_2_O_3_ promoter was doped, the deposition of carbon was diminished. For instance, for 1.0 wt % Ho_2_O_3_, the weight loss was 15.0%, while for 2.0, 3.0, 4.0, and 5.0 wt % Ho_2_O_3_ loadings, the weight loss was quantified as 13, 22, 14, and 13%, respectively. These findings suggested that the addition of Ho_2_O_3_ favored the stability of the catalyst and reduced the formation of carbon.

## Conclusions

The current study comprehended the development of 5%Ni-*x*%Ho-8%YZr catalysts (*x* = 0.0, 1.0, 2.0, 3.0, 4.0, or 5.0) through the impregnation technique for CRM. The investigation focused on the impact of the Ho_2_O_3_ promoter content on the role of yttria-stabilized cubic zirconia-supported nickel-based catalysts in terms of the conversion of the reactants. The addition of the Ho_2_O_3_ promoter enhanced the average CH_4_ conversion. When Ho_2_O_3_ loading was increased from 0.0 to 1.0, 2.0, 3.0, and 4.0 wt %, the acquired average conversions were 73, 76, 78, 79, and 85%, respectively. The promoter increased the H_2_/CO mole ratio, that is, increased the hydrogen production via carbon dioxide binding to Ho^3+^ ions and forming radical carbonate anions, which, in turn, facilitated the activation of terminal C=O bonds, and hence, methane decomposition by the generated surface oxygen radical and by the assistance of catalyst surface acidity. In addition, the promoter strengthened the interaction between nickel oxide and the yttria-stabilized zirconia support, which contributed to the prevention of sintering the nickel particles and therefore assisted the very good catalytic performance. The addition of the promoter increased the crystallite size for the fresh catalysts due its incorporation in the lattice of the yttria-stabilized zirconia support, while reduction in crystallite size for the spent catalyst was observed owing to the expelling of the promoter from the lattice of the support. Such an ability of inserting and exerting the promoter in and from the lattice of the support gave another tool to prevent catalyst deactivation by sintering. The Ni-4Ho-YZr catalyst gave the best achievment in activity and stability. The N_2_-physisorption adsorption–desorption isotherms pointed out that the BET specific surface area results were within the range of 25.7–33.3 m^2^/g, pore volume in the range of 0.15–0.21 g/cm^3^, and pore size in the range of 23.5–24.9 nm, indicating that loading of the promoter had a marginal effect on these textural properties. The optimum loading of the Ho_2_O_3_ promoter was 4.0 wt % because the Ni-4Ho-YZr catalyst depicted the maximum activity toward CRM under the stipulated operating conditions. The H_2_-TPR results indicated that the amount of hydrogen consumption was reduced by the amount of Ho_2_O_3_ promoters from 4 to 0%. The TGA analysis denoted that the amount of deposited carbon on the Ho_2_O_3_-promoted catalyst was less than that of the un-promoted one.

## Supporting Information Available

Catalyst characterization, catalyst performance test, and catalyst constituent (PDF)

## Notes

^⊥^ First Co-authors.
