# Supplementary material for: Effect of Holmium Oxide Loading on Nickel Catalyst Supported on Yttria-Stabilized Zirconia in Methane Dry Reforming
Source: ACS Omega. 2022 Nov 21;7(48):43700–9. doi: 10.1021/acsomega.2c04320 (PMC9730481; doi:10.1021/acsomega.2c04320)
Supplement: Supplementary file 1 — ao2c04320_si_001.pdf [file ao2c04320_si_001.pdf]

SUPPORTING INFORMATION

Effect of Holmium Oxide loading on Nickel Catalyst Supported on Yttria–stabilized Zirconia in Methane Dry Reforming

Ahmed Sadeq Al-Fatesh[*1a], Ahmed A. Ibrahim[1], Anis H. Fakeeha[1], Fahad Albaqi[2a], Khalid Anojaidi[2], Ibrahim Albinali[2], Ahmed E. Abasaeed[1], Francesco Frusteri[3], Sofiu L. Mahmud[1], Jehad K. Abu-Dahrieh[*4a], Abdulaziz A. Bagabas[*2]

[1] Chemical Engineering Department, College of Engineering, King Saud University, P.O. Box 800, Riyadh 11421, Saudi Arabia

[2] National Petrochemical Technology Center (NPTC), Materials Science Research Institute (MSRI), King Abdulaziz City for Science and Technology (KACST), P.O. Box 6086, Riyadh 11442, Saudi Arabia

[3] CNR-ITAE, Istituto di Tecnologie Avanzate per Energia "Nicola Giordano", Via S. Lucia sopra Contesse 5, 98126, Messina, Italy

[4] School of Chemistry and Chemical Engineering, Queen's University Belfast, Belfast BT9 5AG, UK

[a] First Co-authors

*Correspondence: aalfatesh@ksu.edu.sa; j.abudahrieh@qub.ac.uk; abagabas@hotmail.com; Tel.: +966-504158546; +44-28-9097-4603; +966-11-481-3790

S1. Catalyst Characterization.

The catalyst characterizations were performed by using various experimental equipment. The specific surface areas of the catalysts were performed by N_2-physisorption at −197 °C. A Micromeritics Tristar II 3020-unit (Micromeritics, Atlanta, GA, USA) was used to obtain the surface area by the standard Brunauer−Emmett−Teller (BET) method. Thermogravimetric analysis in atmospheric air via a TGA-15 SHIMADZU analyzer (Shimadzu Corporation, Kyoto, Japan) was employed to compute the extent of carbon formation on the surface of spent catalysts. The morphology of the catalyst samples was examined by using a high-resolution transmission electron microscope (HRTEM model:

JEM-2100 F, JEOL, Akishima, Tokyo, Japan) and a field-emission scanning electron microscope (FE-SEM, 7100F JEOL, Tokyo, Japan), equipped with energy-dispersive X-ray spectroscopy (EDX) for surface elemental analysis. The textural properties were investigated by temperature-programmed desorption of CO_2 (CO_2-TPD) and H_2 temperature programmed reduction (H_2-TPR) by using Micromeritics AutoChem II, Atlanta, GA, USA. The phase identification and crystallite size were determined by powder X-ray diffraction (Rigaku, Tokyo, Japan).

S2. Catalyst performance test

The CO_2 reforming of methane was performed by using a stainless steel fixed-bed reactor (PID Eng. & Tech Micro activity Reference Company) having 9.1 mm internal diameter and 300 mm height, operated at 1.0 atm. The activation of 0.1 g of catalyst was carried out under 1200 mL of H_2 flow for 1.0 h at 700 ^{o}C. Then after eliminating the physiosorbed H_2, the N_2 treatment was performed for 15 minutes. The volume ratio of $CO_2/CH_4/N_2$ was set to 3/3/1 at a 4.2 L/h flow rate for generating a 42 L/h/g_{cat} of gas hourly space velocity (GHSV). To determine feed and output gas compositions, a thermal conductivity detector of "GC-2014 SHIMADZU" was used. After cooling the reactor by N_2 gas, the characterizations of the spent catalysts were performed. The conversion expressions are stated as:

$$\mathrm{CH_4\ conversion\ (\%)} = \frac{\mathrm{CH_{4,in}} - \mathrm{CH_{4,out}}}{\mathrm{CH_{4,in}}} \times 100$$

$$\mathrm{CO_2\ conversion\ (\%)} = \frac{\mathrm{CO_{2,in}} - \mathrm{CO_{2,out}}}{\mathrm{CO_{2,in}}} \times 100$$

S3 Catalyst constituent

For 1.0 g of catalyst and 1.0 wt.% Ho_2O_3 promoter, the amounts used were 0.2476g of Ni $(NO_3)_2{\cdot}6H_2O$; 0.03g of Ho $(NO_3)_3{\cdot}5H_2O$, and 0.7224g of *meso*-$8Y_2O_3$-ZrO_2 support.

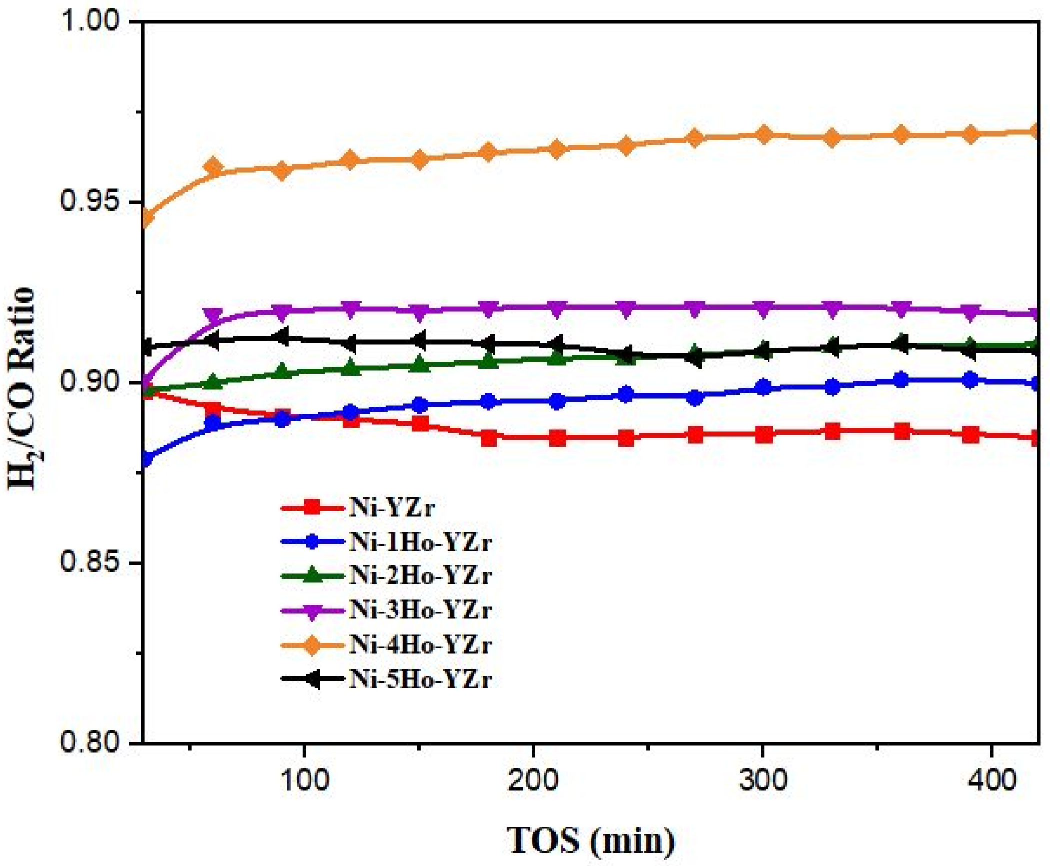

Figure S1. H_2/CO ratio versus time-on-stream.

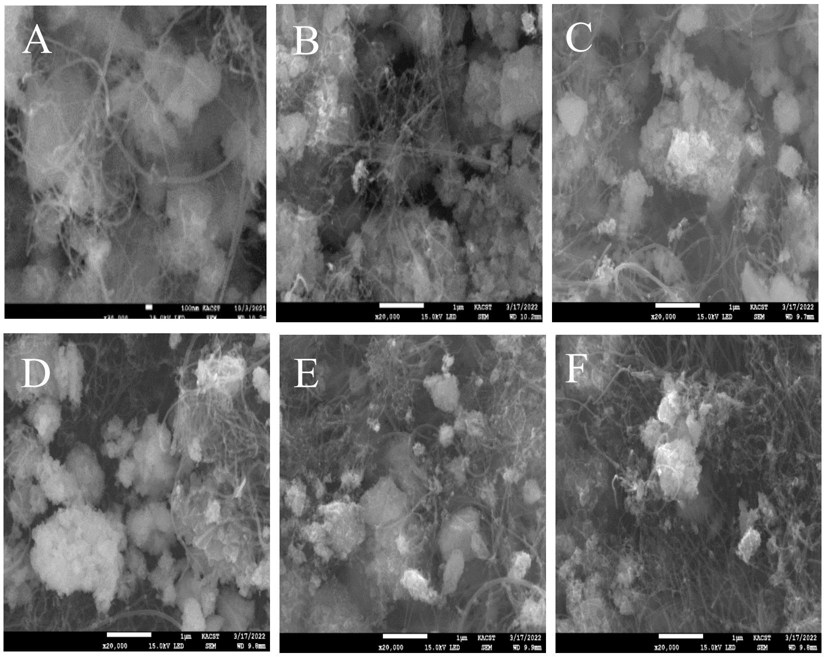

Figure S2. SEM images of the spent catalysts: (A) Ni-0Ho-YZ, (B) Ni-1Ho-YZ, (C) Ni-2Ho-YZ, (D) Ni-3Ho-YZ, (E) Ni-4Ho-YZ, and (F) Ni-5Ho-YZ.

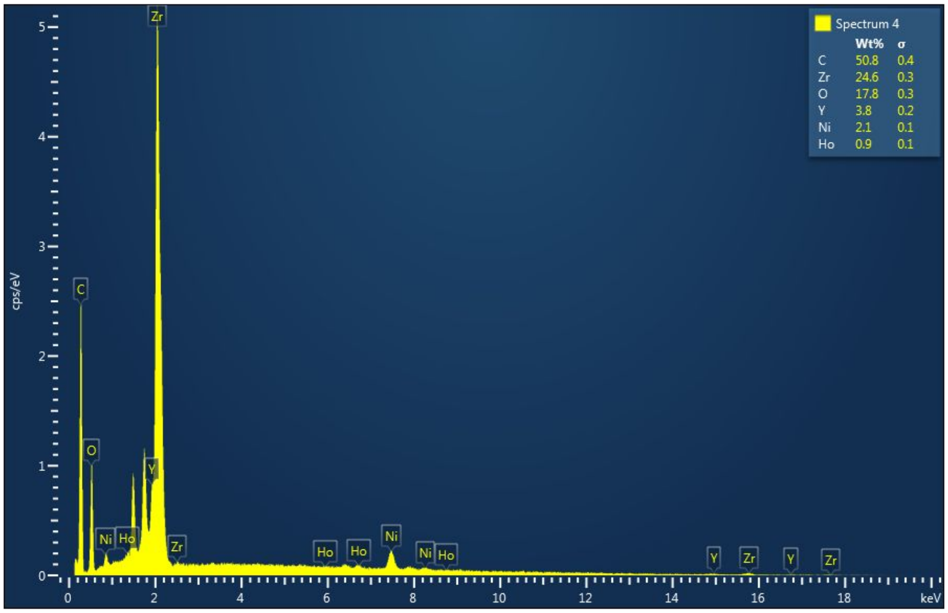

Figure S3. EDX of the spent Ni-4Ho-YZ catalyst.

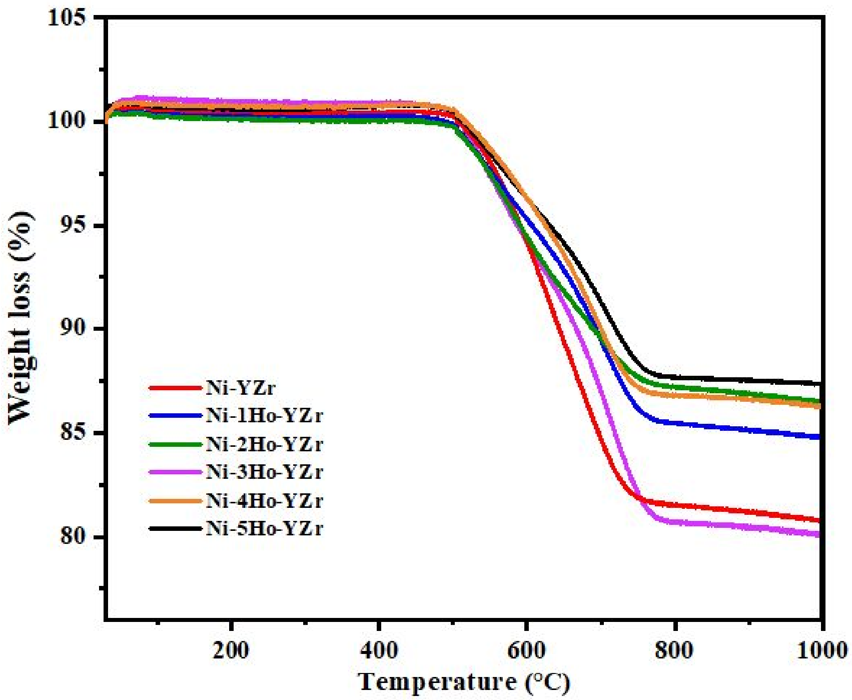

Figure S4. TGA profiles for the Ni-xHo-YZr (x = 0, 1, 2, 3, 4, 5) catalysts after 7.0 hours in DRM, operated at 800 °C and 1.0 atmosphere.
